# Alcohol misuse, health-related behaviors, and burnout among clinical therapists in China during the early Covid-19 pandemic: A Nationwide survey

**DOI:** 10.3389/fpubh.2023.1084259

**Published:** 2023-04-06

**Authors:** Rui Tao, Michael Hsu, Kaiyuan Min, Daming Mo, Feng Geng, Lei Xia, Tingfang Liu, Yuanli Liu, Feng Jiang, Huanzhong Liu, Yi-lang Tang

**Affiliations:** ^1^Department of Substance-Related Disorders, Affiliated Psychological Hospital of Anhui Medical University, Hefei, China; ^2^Department of Psychiatry, Chao hu Hospital of Anhui Medical University, Hefei, China; ^3^Department of Psychiatry, School of Mental Health and Psychological Sciences, Anhui Medical University, Hefei, China; ^4^Department of Substance-Related Disorders, Hefei Fourth People’s Hospital, Hefei, China; ^5^Addiction Psychiatry Fellowship Program, Department of Psychiatry and Behavioral Sciences, Emory University, Atlanta, GA, United States; ^6^State Key Laboratory of Medical Molecular Biology, Institute of Basic Medical Sciences, Chinese Academy of Medical Sciences and Peking Union Medical College, Beijing, China; ^7^Department of Psychiatry, Second Affiliated Hospital of Anhui Medical University, Hefei, China; ^8^Research Department, School of Health Policy and Management, Chinese Academy of Medical Sciences & Peking Union Medical College, Beijing, China; ^9^Research Department, School of International and Public Affairs, Shanghai Jiao Tong University, Shanghai, China; ^10^Research Department, Institute of Healthy Yangtze River Delta, Shanghai Jiao Tong University, Shanghai, China; ^11^Department of Psychiatry, Anhui Psychiatric Center, Hefei, China; ^12^Mental Health Service Line, Atlanta Veterans Affairs Medical Center, Decatur, GA, United States

**Keywords:** alcohol use, alcohol misuse, clinical therapists, COVID-19 pandemic, risk factor

## Abstract

**Objectives:**

This study aimed to assess the extent of alcohol use and misuse among clinical therapists working in psychiatric hospitals in China during the early COVID-19 Pandemic, and to identify associated factors.

**Methods:**

An anonymous nationwide survey was conducted in 41 tertiary psychiatric hospitals. We collected demographic data as well as alcohol use using the Alcohol Use Disorders Identification Test-Consumption (AUDIT-C) and burnout using the Maslach Burnout Inventory Human Services Survey.

**Results:**

In total, 396 clinical therapists completed the survey, representing 89.0% of all potential participants we targeted. The mean age of participants was 33.8 years old, and more than three-quarters (77.5%) were female. Nearly two-fifths (39.1%) self-reported as current alcohol users. The overall prevalence of alcohol misuse was 6.6%. Nearly one-fifth (19.9%) reported symptoms of burnout with high emotional exhaustion in 46 (11.6%), and high depersonalization in 61 (15.4%). Multiple logistic regression showed alcohol use was associated with male gender (OR = 4.392; 95% CI =2.443–7.894), single marital status (OR = 1.652; 95% CI =0.970–2.814), smoking habit (OR = 3.847; 95%CI =1.160–12.758) and regular exercise (OR = 2.719; 95%CI =1.490–4.963). Alcohol misuse was associated with male gender (OR = 3.367; 95% CI =1.174–9.655), a lower education level (OR = 3.788; 95%CI =1.009–14.224), smoking habit (OR = 4.626; 95%CI =1.277–16.754) and high burnout (depersonalization, OR = 4.848; 95%CI =1.433–16.406).

**Conclusion:**

During the COVID-19 pandemic, clinical therapists’ alcohol consumption did not increase significantly. Male gender, cigarette smoking, and burnout are associated with an increased risk of alcohol misuse among clinical therapists. Targeted intervention is needed when developing strategies to reduce alcohol misuse and improve clinical therapists’ wellness and mental health.

## Introduction

The COVID-19 outbreak pandemic had a significant impact on the physical and mental health of people across the world (1, 2). Several studies demonstrated that alcohol use/misuse and other mental health problems increased with the onset of the COVID-19 pandemic (3–6). An online survey of 1,118 U.S. adults showed that there was a disproportionate increase in alcohol-related health problems among male adults, further mediated by drinking motives and social stressors (3). The COVID-19 pandemic contributed to psychological distress and burnout among healthcare workers (7–9). Front-line healthcare workers may use alcohol or other substances to cope with negative moods and stress (10, 11). Substance use was also a risk factor for increased psychological distress (8), which could lead to poorer mental and physical well-being (4, 12, 13). Different samples (occupational characteristics), working environment, work intensity, and stress level during the epidemic may be risk factors for increased alcohol consumption during the COVID-19 pandemic. Healthcare workers have reported high levels of burnout, and additional stressors related to the pandemic may further accelerate burnout (14), which may affect alcohol use and misuse among healthcare workers (15, 16).

Alcohol consumption among healthcare workers is an important area of research. Many healthcare workers play an essential role in the prevention, screening, and management of substance use disorders. Alcohol use and misuse among healthcare workers may negatively impact their psychological and physical well-being as well as patient care (17). Several recent studies from different countries showed that healthcare workers are also at risk for hazardous alcohol consumption. A cross-sectional study in the UK showed that occupational distress and work-related factors can increase physicians’ alcohol consumption (18). During the pandemic, clinical therapists have had to carry the dual burden of addressing the extraordinary distress that their patients face in addition to coping with their own struggles. Yet, data describing clinical therapists’ alcohol use and misuse is scarce.

Clinical therapists are a relatively new profession in China. According to the Chinese professional standards and certification system, clinical therapists’ educational and training backgrounds are diverse, and they can be psychologists, social workers, or nurses as long as they have received the required training and are certified. The role of clinical therapists in China is to provide psychological counseling, therapy, and education to patients with mental disorders (19, 20). To the best of our knowledge, the present study is the first to examine the current prevalence of alcohol use/misuse and identify the risk factors that contribute to alcohol use/misuse among Chinese clinical therapists during the early COVID-19 pandemic. We hypothesized that clinical therapists would be at increased risk of alcohol misuse and use during the pandemic. A primary aim of our study is to inform policymakers and institutional leadership regarding the scope of alcohol use and misuse among healthcare workers to hopefully encourage and inform treatment interventions.

## Methods

### Study design, setting, and participants

We used cross-sectional data from a multicenter, nationally representative survey of clinical therapists in China, which was collected anonymously through WeChat from January 2021 to March 2021. We adopted whole-group sampling to investigate alcohol use and misuse among clinical therapists and their risk factors during the early Covid-19 pandemic. All clinical therapists (*N* = 445) from 41 tertiary psychiatric hospitals in 29 provinces in China were invited to participate in the survey. Excluding the unfinished questionnaires and the questionnaires with logical problems, a total of 396 questionnaires were finally included in the statistical analysis (the response rate was 89.0%).

In this cross-sectional study, Socio-demographic characteristics (gender, age, education levels, marital status, income), health-related behaviors (smoking or not, insomnia, regular physical exercise), alcohol use/misuse, and occupational burnout were collected with the online questionnaire. AUDIT-C was used to investigate the patterns of alcohol use, and Maslach Burnout Inventory Human-Services-Survey (MBI-HSS) was used to investigate their occupational burnout.

### Questionnaire

We developed the electronic questionnaire based on existing literature and prior studies (16, 21). AUDIT-C is a concise version of the AUDIT and was used to assess patterns of alcohol use among clinical therapists. A higher total score (ranging from 0 to 12) is associated with higher severity of alcohol use problems. Through the AUDIT-C scores, the participants were divided into probable alcohol misuse [a total score of ≥3 (women) and ≥ 4 (men)] and low-risk users of alcohol [a total score of <3 (women) and < 4 (men)] (22, 23).

Occupational burnout was assessed using the MBI-HSS, which is a psychological assessment instrument including 22 items about occupational burnout that includes three subscales: emotional exhaustion (EE, which assesses the experience of being emotionally exhausted by the demands of work), depersonalization (DP, refers to the degree to which each one recognizes attitudes of unfeeling and impersonal response from the recipients of their care) and personal accomplishment (PA, refers to the feelings of self-efficacy and accomplishment at work). We considered the cut-off of EE ≥ 27 points or DP ≥ 10 points to define the presence of occupational burnout following previous studies (24).

### Statistical analysis

Data analysis was performed using IBM’s Statistical Package of Social Sciences (SPSS version 22.0). The sample distribution was conducted using frequency for categorical variables and mean ± standard deviation for continuous variables. For the statistical analysis, the Chi-square test was utilized to assess the variables which were not in the normal distribution. The independent correlates of current alcohol use and alcohol misuse were examined by multiple logistic regression, and alcohol use or alcohol misuse were the dependent variables (yes = 1, no = 0). Age groups (≤34 years old, 35–49 years old, or ≥ 50 years old), educational level (associate degree or less, college degree, master’s degree or more), gender (male and female); marital status (single, married, divorced/widowed); smoking habit (yes/no); and other studied variables were entered as the independent variables. The level of statistical significance was set at *value of p*s of 0.05 (two-tailed).

## Results

### Socio-demographic characteristics of clinical therapists in China

The socio-demographic characteristics of our sample were shown in Table 1. The mean age of participants was 33.8 years old. 307 subjects (77.5%) were female, more than two-thirds (67.2%) were younger than 35, and 62.4% were married. 155 participants (39.1%) self-reported as current alcohol users. Significant differences were found between participants who did and did not report alcohol with respect to the demographic (gender, education, marital status) and health-related behaviors (smoking habit, regular exercise). Significant differences were found between those with and without alcohol misuse concerning the demographic (gender, education, smoking habit) and burnout factors (depersonalization) (all *p*< 0.05) (Table 1). Nearly one-fifth (19.9%) of responding clinical therapists reported symptoms of burnout with high emotional exhaustion in 46 (11.6%), and high depersonalization in 61 (15.4%). Among 155 current alcohol users, 41 (26.5%) reported alcohol use less than before, 103 participants (66.5%) reported no change, and 11 (7.1%) reported an increase in alcohol use than before. Participation in the front-line work of treating COVID-19 was not significantly associated with alcohol use and misuse among Chinese clinical therapists.

**Table 1:** Socio-demographic Characteristics and burnout of clinical therapists in China (*N* = 396).

Variables	Participants	Alcohol use		Alcohol misuse		Alcohol use	Alcohol misuse
	*n* = 396	Yes (*n* = 155)	No (*n* = 241)	Yes (*n* = 26)	No (*n* = 370)	χ2 (*value of p*)	χ2 (*value of p*)
Age group (*N*, %)	33.86 ± 7.62	34.05 ± 8.20	33.74 ± 7.24	35.50 ± 8.35	33.75 ± 7.56		
≤34	266 (67.2)	106 (39.8)	160 (60.2)	14 (5.3)	252 (94.7)		
35–49	105 (26.5)	38 (36.2)	67 (63.8)	10 (9.5)	95 (90.5)		
≥50	25 (6.3)	11 (44.0)	14 (56.0)	2 (8.0)	23 (92.0)	0.688 (0.709)	2.317 (0.314)
*Gender (N, %)*							
Male	89 (22.5)	63 (70.8)	26 (29.2)	15 (16.9)	74 (83.1)		
Female	307 (77.5)	92 (30.0)	215 (70.0)	11 (3.6)	296 (96.4)	48.262 (<0.001)	19.808 (<0.001)
*Education (N, %)*							
Bachelor degree or less	241 (60.9)	105 (43.6)	136 (56.4)	23 (9.5)	218 (90.5)		
Master degree or more	155 (39.1)	50 (32.3)	105 (67.7)	3 (1.9)	152 (98.1)	5.066 (0.024)	8.901 (0.003)
*Marital status (N, %)*							
Married	247 (62.4)	84 (34.0)	163 (66.0)	15 (6.1)	232 (93.9)		
Single	134 (33.8)	63 (47.0)	71 (53.0)	9 (6.7)	125 (93.3)		
Divorced or widowed	15 (3.8)	8 (53.3)	7 (46.7)	2 (13.3)	13 (86.7)	7.488 (0.024)	1.223 (0.543)
*Smoking habit (N, %)*							
No	371 (93.7)	134 (36.1)	237 (63.9)	18 (4.9)	353 (95.1)		
Yes	25 (6.3)	21 (84.0)	4 (16.0)	8 (32.0)	17 (68.0)	22.542 (<0.001)	28.140 (<0.001)
*Income (N, %)*							
Low (≤5000RMBs)	101 (25.5)	33 (32.7)	68 (67.3)	5 (5.0)	96 (95.0)		
Medium (5000-10000RMs)	194 (49.0)	87 (44.8)	107 (55.2)	16 (8.2)	178 (91.8)		
High (≥10,000 RMs)	101 (25.5)	35 (34.7)	66 (65.3)	5 (5.0)	96 (95.0)	5.278 (0.071)	1.753 (0.416)
*Frequent insomnia (N, %)*							
No	111 (28)	40 (36.0)	71 (64.0)	7 (6.3)	104 (93.7)		
Yes	285 (72)	115 (40.4)	170 (59.6)	19 (6.7)	266 (93.3)	0.624 (0.429)	0.017 (0.897)
*Regular exercise (N, %)*							
No	97 (24.5)	22 (22.7)	75 (77.3)	5 (5.2)	92 (94.8)		
Yes	299 (75.5)	133 (44.5)	166 (55.5)	21 (7.0)	278 (93.0)	14.613 (<0.001)	0.417 (0.518)
*Emotional Exhaustion (N, %)*							
No	350 (88.4)	138 (39.4)	212 (60.6)	22 (6.3)	328 (93.7)		
Yes	46 (11.6)	17 (37.0)	29 (63.0)	4 (8.7)	42 (91.3)	0.104 (0.747)	0.385 (0.535)
*Depersonalization (N, %)*							
No	335 (84.6)	131 (39.1)	204 (60.9)	17 (5.1)	318 (94.9)		
Yes	61 (15.4)	24 (39.3)	37 (60.7)	9 (14.8)	52 (85.2)	0.001 (0.972)	7.881 (0.005)
*Personal Accomplishment (N, %)*							
Low	156 (39.4)	60 (38.5)	96 (61.5)	11 (7.1)	145 (92.9)		
High	240 (60.6)	95 (39.6)	146 (60.4)	15 (6.3)	225 (93.8)	0.050 (0.823)	0.099 (0.753)
*Participated in the frontline work of COVID-19 (N, %)*							
No	313 (79.0)	120 (38.3)	193 (61.7)	22 (7.0)	291 (93.0)		
Yes	83 (21.0)	35 (42.2)	48 (57.8)	4 (4.8)	79 (95.2)	0.404 (0.525)	0.522 (0.470)

### Alcohol use and misuse and related factors among clinical therapists

Less than one-third (30.3%) of all participants reported consuming alcohol once a month or less, 6.3% reported at least twice a month, and 2.5% reported twice a week or more. More than two-thirds (68.4%) of all current alcohol users were in the age group of ≤34 years old, and 53.8% of all alcohol misusers were ≤ 34 years old. The prevalence of alcohol misuse was 6.6% overall and there was a significant gender difference (16.9% in males and 3.6% in females, *p* < 0.001). The overall prevalence of alcohol use among the participants was 39.1% with a significant gender difference (70.8% prevalence in males and 30% in females, *p* < 0.01. Compared to those with Master’s degrees or more (39.1%), those with Bachelor’s degrees or less (60.9%) had significantly higher rates of alcohol use and misuse (both *p* < 0.01). The smoking rate among alcohol misusers was more than 5 times higher than those who did not report alcohol misuse (30.8% vs. 4.6%, *p* < 0.001). The smoking rate among alcohol users was higher than that of non-alcohol misusers (13.5% vs. 1.7%, *p* < 0.01). The rate of alcohol use was significantly higher in people with regular exercise (defined as exercising at least three times per week in the past month according to the recommendations of the National Fitness Guideline) than in those without regular exercise (44.5% vs. 22.7%, *p* < 0.01). Alcohol misuse was higher among clinical therapists who reported high job burnout (depersonalization) than those who did not report such burnout (14.8% vs. 5.1%, *p* < 0.01) (Table 1).

### Factors associated with alcohol use and misuse in a multiple logistic regression

We divided the samples (*N* = 396) into current alcohol users (those who drank alcohol in the past 12 months; *N* = 155) and non-alcohol users (*N* = 241) and performed multiple logistic regression analyses to examine the associations between alcohol use and other factors. The references of the categorical variables were defined as shown in Table 2. Alcohol use among clinical therapists was associated with male gender (OR = 4.392; 95% CI =2.443–7.894), marital status (single, OR = 1.652; 95% CI =0.970–2.814), smoking habit (OR = 3.847; 95%CI =1.160–12.758) and regular exercise (OR = 2.719; 95%CI =1.490–4.963).

**Table 2:** Multiple logistic regression examining individual characteristics associated with alcohol use in Chinese clinical therapists.

Variables	B	*p-*value	OR	95% CI
*Age group (ref. ≤34 years old)*				
35–49	−0.014	0.965	0.986	0.539–1.807
≥50	−0.314	0.541	0.731	0.267–1.998
*Gender (ref. Female)*				
Male	1.480	<0.001	4.392	2.443–7.894
*Education (ref. Master degree or more)*				
Bachelor degree or less	0.391	0.119	1.479	0.904–2.418
*Marital status (ref. Married)*				
Single	0.502	0.045	1.652	0.970–2.814
Divorced or widowed	1.126	0.055	3.082	0.975–9.746
*Smoking habit (ref. No)*				
Yes	1.347	0.028	3.847	1.160–12.758
Income (ref. Low)				
Medium	0.471	0.100	1.601	0.914–2.804
High	0	1.000	1.000	0.502–1.991
*Frequent insomnia (ref. No)*				
Yes	0.303	0.251	1.354	0.807–2.272
*Regular exercise (ref. No)*				
Yes	1.000	0.001	2.719	1.490–4.963
*Emotional Exhaustion (ref. No)*				
Yes	−0.083	0.847	0.920	0.396–2.138
*Depersonalization (ref. No)*				
Yes	−0.264	0.494	0.768	0.361–1.636
*Personal Accomplishment (ref. High)*				
Low	0.053	0.829	1.055	0.650–1.711
*Participated in the frontline work of COVID-19 (ref. No)*				
Yes	0.164	0.565	1.179	0.673–2.063

We also used AUDIT-C cut-off scores (alcohol misuse: the AUDIT-C score of ≥3 (women) and ≥ 4 (men)) to divide participants into those with probable alcohol misuse (*N* = 26) and those without (*N* = 370). Multiple logistic regression was used to examine the association between probable alcohol misuse and other variables (Table 3). A higher risk of alcohol misuse was associated with male gender (OR = 3.367; 95% CI =1.174–9.655), low education level (OR = 3.788; 95%CI =1.009–14.224), smoking habit (OR = 4.626; 95%CI =1.277–16.754) and high burnout (depersonalization, OR = 4.848; 95%CI =1.433–16.406).

**Table 3:** Multiple logistic regression examining individual characteristics associated with alcohol misuse in Chinese clinical therapists.

Variables	*B*	*p-*value	OR	95% CI
*Age group (ref. ≤34 years old)*				
35–49	0.936	0.131	2.551	0.757–8.589
≥50	0.308	0.747	1.360	0.210–8.816
*Gender (ref. Female)*				
Male	1.214	0.024	3.367	1.174–9.655
*Education (ref. Master degree or more)*				
Bachelor degree or less	1.332	0.048	3.788	1.009–14.224
*Marital status (ref. Married)*				
Single	0.225	0.700	1.252	0.399–3.934
Divorced or widowed	1.483	0.112	4.404	0.707–27.418
*Smoking habit (ref. No*)				
Yes	1.532	0.020	4.626	1.277–16.754
*Income (ref. Low)*				
Medium	0.251	0.664	1.286	0.415–3.987
High	−0.535	0.505	0.586	0.122–2.823
*Frequent insomnia (ref. No)*				
Yes	−0.340	0.528	0.712	0.248–2.046
*Regular exercise (ref. No)*				
Yes	0.454	0.445	1.574	0.491–5.047
*Emotional Exhaustion (ref. No)*				
Yes	−0.336	0.646	0.715	0.171–2.991
*Depersonalization (ref. No)*				
Yes	1.579	0.011	4.848	1.433–16.406
*Personal Accomplishment (ref. High)*				
Low	0.040	0.936	1.041	0.392–2.763
*Participated in the frontline work of COVID-19 (ref. No)*				
Yes	−0.400	0.516	0.670	0.200–2.241

## Discussion

To our knowledge, no prior studies have assessed alcohol use and misuse among clinical therapists in China during the COVID-19 pandemic. We found alcohol misuse was not uncommon among Chinese clinical therapists, with 6.6% reporting probable alcohol misuse and 39.1% reporting general alcohol use. We also found that nearly one-fifth (19.9%) of Chinese clinical therapists reported high levels of occupational burnout. Alcohol misuse was significantly associated with male gender, having a bachelor’s degree or less, smoking habits, and high occupational burnout (depersonalization). Somewhat surprisingly, we found that working on the frontlines of treating COVID-19 was not significantly associated with either alcohol use or misuse. Additionally, unlike results from prior works, only 7.1% of individuals reported an increase in alcohol use during the COVID-19 pandemic while 26.5% of individuals reported a decrease in alcohol use and the majority of individuals (66.5%) reported that the pandemic had no significant impact on their alcohol consumption. The existing literature on this is mixed. Some studies reported an increase in alcohol consumption (25), while others showed that healthcare workers’ alcohol consumption decreased significantly during the COVID-19 pandemic (16).

There was no significant increase in alcohol use among clinical therapists during the COVID-19 pandemic according to our study. One potential explanation is that clinical therapists are better equipped to cope with negative emotions due to the nature of their profession (26, 27). Another potential explanation could be that individuals frequented liquor stores and other social drinking events during the COVID-19 pandemic due to social distancing (16, 28). Nonetheless, occupational burnout remains an important issue for clinical therapists.

### Socio-demographics, health-related behaviors and alcohol use/misuse

Some common variables were significantly associated with both alcohol use and alcohol misuse among clinical therapists, such as smoking habits (OR = 3.847 and 4.626 in current alcohol users and alcohol misusers, respectively) and male gender (OR = 4.392 and 3.367 in current alcohol users and alcohol misusers, respectively). Lower education level (bachelor’s degree or less) and occupational burnout (depersonalization) were significantly associated only with alcohol misuse, while marital status (single) and regular exercise were significantly associated only with alcohol use.

### Male gender, smoking habits and alcohol use/misuse

The results of this study reported rates of alcohol use and misuse that were significantly higher in males (70.8 and 16.9%, respectively) compared with females (30.0 and 3.6%, respectively). In the multivariate analysis, male clinical therapists were more likely to be alcohol users (OR = 4.392; 95% CI =2.443–7.894) and misusers (OR = 3.367; 95% CI =1.174–9.655) than female therapists. Consistent with previous studies (29–32), male gender was a significant risk factor for alcohol use and alcohol misuse. A recent study of mental health professionals in 41 hospitals in China (3,479 males and 10,501 females) also found significant differences between male and female medical workers in alcohol misuse with nearly two-thirds (63.3%) of alcohol misusers being male (21).

We also found a significant association between smoking habits and alcohol consumption. Cigarette use was significantly associated with reported alcohol misuse (OR = 4.626; 95%CI =1.277–16.754) and alcohol use (OR = 3.847; 95%CI =1.160–12.758). Our finding about the association between alcohol use/misuse and cigarette use among clinical therapists is consistent with findings from several other studies conducted in China (33, 34) and other countries (29, 35–37). The combined use of alcohol and tobacco have a multiplicative effect on the risk of health problems (38–40). Tverdal et al. (41) found that an association between higher levels of alcohol consumption (more than one alcohol glass/day) and an increased risk of pancreatic cancer could be explained by tobacco use. Some studies showed that helping patients quit and reduce smoking may reduce their alcohol use/misuse (35, 42). Therefore, addressing tobacco use could also be a way to decrease the risk of alcohol use/misuse among clinical therapists.

### Marital status, regular physical exercise, and alcohol use

Our survey found that single marital status and regular physical exercise were risk factors for alcohol use among clinical therapists. 75.5% of clinical therapists in our sample reported exercising regularly. A positive association between exercise and alcohol use is also found in prior studies of non-clinical individuals across the world, potentially explained by social factors, personality factors, and shared reward response pathways in the mesocorticolimbic region. The positive relationship between single marital status and drinking may be explained by increased engagement in social drinking (e.g., going to bars; drinking after regular exercise) among unmarried participants (43). Consistent with prior studies, a stable marital relationship may also reduce the risk of medical staff’s alcohol use and alcohol misuse (21). Compared to never-married individuals, divorced/separated individuals were significantly more likely to report binge drinking in the past year (44). However, the results of our survey do not suggest a significant association between divorced or widowed and alcohol use and misuse, which may be due in part to the specific characteristics of clinical therapists such as their ability to cope with and process through challenging situations.

### Education level, burnout, and alcohol misuse

A low education level (bachelor’s degree or less) was a risk factor for alcohol misuse among clinical therapists. Studies from other countries also suggest that education level may affect perception and use of alcohol. Individuals that attained lower education levels may consume more alcohol than those that attained higher education levels (45). Previous studies showed that healthcare workers with higher education levels might have a higher risk awareness regarding alcohol use/misuse and are less likely to misuse alcohol. However, two studies found the opposite. One study in Austria involving 400 office-based physicians showed that physicians with higher education admitted to drinking alcohol more frequently (46). Another recent study of 13,980 medical professionals in China found that those with high education levels drink more heavily than those with lower education levels, especially among doctors (21). One possible explanation is that a higher level of education usually means more disposable money and a possibly broader social network, which may in turn contribute to drinking (21, 47).

Similar to the results of previous studies (9, 48–51), occupational burnout among healthcare workers was at a relatively high level (19.9%). In our study, occupational burnout (depersonalization) was associated with an increased risk of alcohol misuse. Alcohol consumption may be a coping strategy to deal with the occupational burnout related to the COVID-19 pandemic (11). Similarly, a cross-sectional survey of 4,000 randomly selected physicians in Danish found that the association between risky alcohol consumption and alexithymia was partially mediated through depersonalization (15). As risky alcohol use and burnout may independently affect patient safety, there is also a need to stratify which risk factors are most likely to lead to occupational burnout to develop appropriate interventions during the epidemic (14, 50). Recent literature explores the impact of the COVID-19 pandemic on burnout among healthcare workers (9, 17), even among clinical therapists (17), and how it has significantly impacted the mental well-being of healthcare workers overall (7, 52). Healthcare workers have reported high levels of burnout, further exacerbated by effects from the COVID-19 pandemic (14). Stress, emotional distress, individual and psychosocial factors, and personality traits are found to be potential risk factors for burnout (53, 54). The management and prevention of occupational burnout in clinical therapists remain important issues as they may lead to turnover and affect workforce sustainability.

### Limitations

Three limitations should be considered when interpreting the findings of the current study. First, the heterogeneity of tools used to measure occupational burnout and AUDIT-C limited the ability to compare the rate of occupational burnout and alcohol misuse across different studies. Second, as a cross-section survey, we cannot infer the causal relationship between alcohol misuse/use and other factors. Third, outcomes are based on self-reports of the amount of drinking/problematic behaviors as opposed to clinical measures.

## Conclusion

Contrary to our prediction, alcohol consumption among Chinese clinical therapists showed no significant increase during the COVID-19 pandemic. This survey identified that occupational burnout, along with male sex, and cigarette smoking, was associated with alcohol misuse during the epidemic, which may guide the development of alcohol misuse detection and intervention practices for this professional group. However, more research is needed to explore specific factors of occupational burnout underlying drinking behavior among Chinese clinical therapists during the pandemic.

## Data availability statement

The original contributions presented in the study are included in the article/supplementary material, further inquiries can be directed to the corresponding authors.

## Ethics statement

The studies involving human participants were reviewed and approved by the study was approved by the Ethical Committee (IRB) at the Chaohu Hospital of Anhui Medical University (202002-kyxm-02). Written consent was obtained before they accessed the online questionnaire from each participant. The patients/participants provided their written informed consent to participate in this study.

## Author contributions

FJ, HL, and Y**-**lT: study design. RT, TL, YL, KM, DM, FG, LX, and Y**-**lT: collection, analyses, and interpretation of data. RT: drafting the first version of the manuscript. MH and Y**-**lT: critical revision of the manuscript. All authors contributed to the article and approved the submitted version.

## Funding

The work was supported by the National Clinical Key Specialty Project Foundation (CN), and the Beijing Medical and Health Foundation (Grant no. MH180924).

## Conflict of interest

The authors declare that the research was conducted in the absence of any commercial or financial relationships that could be construed as a potential conflict of interest.

## Publisher’s note

All claims expressed in this article are solely those of the authors and do not necessarily represent those of their affiliated organizations, or those of the publisher, the editors and the reviewers. Any product that may be evaluated in this article, or claim that may be made by its manufacturer, is not guaranteed or endorsed by the publisher.
